# “How can we help you?”: results of a scoping review on the perceived needs of people living with chronic pain regarding physiotherapy

**DOI:** 10.1186/s12913-024-11805-3

**Published:** 2024-11-14

**Authors:** Jonathan Gervais-Hupé, Arthur Filleul, Kadija Perreault, Isabelle Gaboury, Timothy H. Wideman, Céline Charbonneau, Fatiha Loukili, Romane Beauvais, Marc-Antoine Campeau, Gevrey Jacob, Noémie Lasnier, Anne Hudon

**Affiliations:** 1 School of rehabilitation, Faculty of medicine, Université de Montréal, PO Box 6128, Centre-Ville, Montreal, QC H3C 3J7 Canada; 2 Centre for Interdisciplinary Research in Rehabilitation of Greater Montreal (CRIR), Institut universitaire sur la réadaptation en déficience physique de Montréal (IURDPM), Centre intégré universitaire de santé et de services sociaux du Centre-Sud-de-l’Ile-de- Montréal (CCSMTL), Montreal, QC Canada; 3 Centre de recherche en éthique (CRÉ), Montreal, QC Canada; 4 Department of bioethics, School of Public Health, Université de Montréal, Montreal, QC Canada; 5 Pragmatic Health Ethics Research Unit, Montreal, QC Canada; 6 Center for Interdisciplinary Research in Rehabilitation and Social Integration (Cirris), Centre intégré universitaire de santé et de services sociaux de la Capitale-Nationale, Quebec City, QC Canada; 7 École des sciences de la réadaptation, Faculté de médecine, Université Laval, Quebec City, QC Canada; 8 Department of family medicine and emergency medicine, Faculty of medicine and health sciences, University of Sherbrooke, Sherbrooke, QC Canada; 9 School of physical and occupational therapy, Faculty of medicine and health sciences, McGill University, Montreal, QC Canada; 10 Centre for Interdisciplinary Research in Rehabilitation of Greater Montreal (CRIR), Centre intégré universitaire de santé et de services sociaux du Centre-Ouest-de-l’Ile-de- Montréal, Montreal, QC Canada; 11 Association québécoise de la douleur chronique, Montreal, QC Canada; 12 Association des personnes vivant avec de la douleur chronique, Gatineau, QC Canada

**Keywords:** Perceived needs, Physiotherapy, Chronic pain

## Abstract

**Background:**

Physiotherapy is effective to reduce pain and improve the quality of life of people living with chronic pain. To offer high-quality physiotherapy services, these services must be patient-centred and respond to patients’ needs. However, few studies seem to target patients’ perceived needs, whereas more studies tend to focus on needs assessed by healthcare experts, which are not always in line with patients’ perceived needs. In addition, people living with chronic pain are often faced with several health inequities and may have varied perceived needs depending on their personal conditions. To offer services that truly meet patients’ needs, it is therefore crucial to understand these needs. This scoping review aims to identify and map the perceived needs of people living with chronic pain towards physiotherapy services.

**Methods:**

To conduct this review, we followed the six stages framework proposed by Arksey and O’Malley. We searched four databases (Medline, Embase, CINHAL and APA PsycINFO) as well as the grey literature. We included all studies describing the needs, demands, preferences or expectations of adults living with chronic pain towards physiotherapy. We then performed an inductive thematic analysis of the results and discussion sections of these studies to identify the perceived needs. Once those needs were identified, we mapped them into the seven dimensions of the patient-centred healthcare delivery framework.

**Results:**

Our review included 96 studies. Various perceived needs were identified through the thematic analysis, such as the needs *for an empathetic relationship*; *for a clear*,* adapted and supervised exercise program;* and *for personalized treatment*. Our mapping into the patient-centred healthcare delivery framework showed that most studies reported needs associated with the dimensions of interpersonal care, individualized healthcare and professional care. Needs associated with the other dimensions of the framework (access; coordination and continuity; services and facilities; data and information) were less frequently mentioned.

**Conclusions:**

The results of this review have enabled us to identify and better understand multiple needs perceived by people living with chronic pain regarding physiotherapy services. The perceived needs identified through this scoping review were mapped within the seven dimensions of the Patient-centred healthcare delivery framework.

**Supplementary Information:**

The online version contains supplementary material available at 10.1186/s12913-024-11805-3.

## Background

Chronic pain affects approximately 20% of the population worldwide [1, 2]. Now recognized as a disease in itself, chronic pain is generally defined as pain lasting longer than the normal expected healing time of tissues (about 3 to 6 months) [3]. Beyond the pain itself, many physical, psychological and social factors are associated with the experience of chronic pain that negatively impact people’s lives [3]. On top of this enormous personal burden, chronic pain generates significant costs for healthcare systems. In 2010, the total yearly cost of pain in the United States ranged from $560 to $635 billion, which is more than the costs of other major diagnoses such as cardiovascular diseases ($309 billion), cancer ($243 billion), and diabetes ($188 billion) [4].

Physiotherapy is among the most frequently used non-pharmacological approaches for chronic pain in the United States [1]. Interventions provided by physiotherapists have been found to be effective in reducing pain intensity and improving the quality of life of people living with chronic pain, among other outcomes [5, 6].

Following recent guidance on patient-centred care [7–9], physiotherapists are encouraged to collaborate with patients and to consider them as unique, while respecting and incorporating their preferences, values and needs in their interventions [10–12]. Despite patients’ needs being a key element to patient-centred care, the term “need” remains a complex and poorly defined term [13–15]. Results of a recent scoping review concluded that studies in the field of rehabilitation, conducted until now, have mainly targeted health related needs based on what was assessed and valued by healthcare representatives and experts, at the expense of needs perceived and expressed by patients [16]. Such attention to the needs evaluated by experts raises many concerns. Focusing on expert-evaluated needs reinforces the presence of a latent paternalism in healthcare frequently decried by many [17]. This paternalism has deleterious effects on patients, thereby limiting their autonomy and freedom to express their own choices and preferences [17]. Moreover, experts’ opinions on the best available interventions are prone to various cognitive biases which can lead to decision-making that dismisses and diverges from patients’ needs [18]. For example, clinicians having sunken cost bias may be tempted to choose an intervention that is not desired by patients, to financially recoup the cost of recently completed training or purchased equipment [18]. Hence, patients’ needs are not always congruent with professionals’ opinions on healthcare needs [19, 20]. In addition to raising ethical issues overlooking the patients’ voice and needs contributes to poor clinical outcomes and low satisfaction with care [21].

Living with chronic pain can be highly challenging. Because chronic pain is invisible and unpredictable [22], people living with this condition often feel misunderstood and stigmatized by healthcare professionals and those around them [23–25]. Their participation in work and social activities can also be greatly affected by their pain [26], and they often end up feeling socially isolated [27]. Moreover, people living with chronic pain often face different types of health inequities [28]. For example, in the United States, non-Caucasian women from lower socio-economic background living in rural areas have been shown to be more prone to have chronic pain [29] and these women’s characteristics were associated with lower use of physiotherapy services in various countries [30]. Several articles also have described the very lengthy and highly limited access to physiotherapy services for people living with chronic pain more generally [31–33]. The multiplicity of challenges faced by people living with chronic pain certainly informs a diversity of perceived needs that are strongly tied to each person’s physical, social, financial, geographical, and psychological condition. Although listening to people living with chronic pain to better respond to their perceived needs is now a prerequisite to offering high quality physiotherapy services, to date, no study has examined the extent and variety of these needs. This scoping review is therefore necessary in order to better identify and understand the diversity of perceived needs of people living with chronic pain with regard to physiotherapy services. Hence, the principal objective of this study was to identify and map the perceived needs of people living with chronic pain towards physiotherapy services.

## Methods

The protocol for this scoping review was previously published [34] and registered on Open Science Framework (registration DOI: 10.17605/OSF.IO/6D8P3). Few modifications have been made since this protocol was published. The exact methodology used to carry out this scoping review is reported here.

We followed the six stages framework for conducting a scoping review proposed by Arksey and O’Malley [35] and enhanced by Levac et al. [36], Daudt et al. [37] and Peters et al. [38]. We also used the Preferred Reporting Items for Systematic reviews and Meta-Analysis-extension for Scoping Reviews checklist (PRISMA-ScR) to guide the reporting of the review (see Appendix 1).

### Stage 1: identifying the research questions

After consultation with all team members, our primary research question asked: What are the perceived needs of people living with chronic pain towards physiotherapy services? Our secondary research question asked: Where are the gaps in the literature on this topic?

### Stage 2: identifying relevant studies

Two librarians helped develop the search strategy. We searched four databases: Medline, Embase, CINHAL and APA PsycINFO (see Appendix 2). No filters were used to limit the results. We also searched the grey literature using Google Scholar, OpenGrey [39] and ProQuest Dissertation & Theses Global (PQDTGlobal) [40]. We then performed a hand search of the reference lists of all the selected full texts to find other relevant references.

All studies presenting or describing the perceived needs of patients with chronic pain towards physiotherapy, regardless of their methodology were included, as well as all studies published in English and French, regardless of their publication date. Conference abstracts were excluded, as they do not provide a sufficient description of patients’ needs in physiotherapy Although reviews were excluded, we examined them to identify and retrieve any studies that had not been previously identified by our search.

#### Definitions of key terms used in the search strategy

##### Patient

The term “patient” in this review related to any adult (18 years old or older) who benefited or who could benefit from physiotherapy services. The term included individuals who wanted to consult in physiotherapy, but who were unable to due to accessibility barriers.

##### Perceived needs

We defined “Perceived needs” as any demands, preferences or expectations from patients towards physiotherapy services, based on their experiences, beliefs and values. We chose this definition because patients’ perceived needs are often associated with patients’ expectations and preferences [41], but there is no established consensus on the definition of the term “needs” regarding physiotherapy or healthcare.

##### Chronic pain

Based on the 11th edition of the International Classification of Diseases (ICD-11) we defined “chronic pain” as a pain lasting longer than 3 months, accompanied by important emotional distress or physical disability [3, 42]. We included studies that discussed any type of chronic pain (chronic primary pain, chronic cancer pain, chronic postsurgical and posttraumatic pain, chronic neuropathic pain, chronic headache and orofacial pain, chronic visceral pain and chronic musculoskeletal pain) [43] to allow to identify a broad range of needs related to physiotherapy services.

##### Physiotherapy services

We included articles describing all the services provided by physiotherapists in any type of healthcare setting (e.g., private clinics; rehabilitation centres, community health centre, home care rehabilitation, etc.). We also included descriptions of patients’ needs regarding any type of interventions in physiotherapy (e.g. clinical treatments in a clinic, YouTube videos, wearable technology, etc.) as long as these were delivered by physiotherapists. We included studies discussing healthcare services other than physiotherapy (e.g., occupational therapy, medicine, psychology, etc.), if patients’ perceived needs regarding physiotherapy were also specifically described. However, we excluded studies describing needs related to multiple healthcare services that did not specifically report on physiotherapy services.

### Stage 3: study selection

We uploaded all identified articles and sources into Covidence, a software specifically designed to manage the conduct of reviews and their study selection (https://www.covidence.org/home). We removed all duplicates and then screened based on titles and abstracts. Two independent reviewers (JGH and AF) first examined a random sample of 50 references to assess the agreement between them and to ensure that the eligibility criteria were relevant and clearly defined. Following this pilot screening, the same two persons reviewed all remaining titles and abstracts. They recorded all the reasons for exclusion. Any discrepancies between reviewers were resolved through discussion. For the second stage of the screening process (full text review), one of the reviewers of the first stage (JGH) and two other independent reviewers (GJ and RB) assessed the remaining studies. Disagreements were also resolved through discussion. One reviewer (GJ) went through all the reviews (systematic or other types of reviews) identified during our literature search to retrieve all studies included in these reviews and relevant to our review that could have been missed. As we wanted to analyse the full scope of results on the topic and aligned with our research questions, all studies meeting the inclusion criteria were included regardless of their quality. The methodological quality or risk of bias of the included studies was not assessed because the purpose of our review was to identify and map the perceived needs in order to outline the breadth of the literature in this field, rather than to evaluate its rigour and reliability [44–46].

### Stage 4: charting the data

Two students (MAC and NL) developed the demographic data extraction sheet using Microsoft Excel (version 16.80). For each study included, the extraction sheet allowed to extract these specific elements: the study characteristics (e.g. title, author’s name, publication date study design, study objectives, etc.), participants’ types of chronic pain (e.g. low back pain, rheumatoid arthritis, knee osteoarthritis, etc.) and the seven dimensions of the “Patient-centred healthcare delivery framework” (see “Stage 5: Collating, summarizing and reporting the results”) discussed in the included studies.

The extraction sheet was then pretested by the same two reviewers (MAC and NL). They extracted the data from two of the included studies using the Excel sheet to ensure the feasibility and quality of the extraction process. In case of disparities, they met to discuss and ensure concordance. They then met with two other reviewers involved in the project (GJ and RB) as well as the first and the senior authors (JGH and AH) to discuss extraction. Minor changes were made to the extraction form.

Following this test, the same four reviewers (GJ, MAC, NL and RB) randomly and equally shared the remaining included articles and extracted their data. Throughout the extraction process, any questions from the reviewers were addressed through discussion or by consulting other members of the team.

### Stage 5: collating, summarising and reporting the results

Since the vast majority of the included studies did not explicitly report and name patients’ perceived needs, we first performed an inductive thematic analysis [47] of the included studies. To do this, we imported the text of the results and discussion sections of the included studies into the QDA miner software [48] and used this software to inductively code these sections. Therefore, segments of the text were associated to a code representing a need. This analysis enabled us to generate perceived needs, as defined above, based on the experiences, expectations and preferences mentioned by the participants of the included studies and/or reported by the authors.

For quantitative studies and the quantitative section of the mixed methods studies, we only analysed the ‘textual’ content from the results and discussion sections, notwithstanding the statistical or numerical components of the results. Once these inductive codes were created, we then used a deductive qualitative approach to coding [49] where we mapped the inductive codes representing patients’ perceived needs to the seven dimensions of the “Patient-centred healthcare delivery framework” proposed by Mühlbacher [50]. This framework was developed to help organize healthcare around patients’ needs [50]. Initially known as the ‘Model of Needs Dimensions’ [51], this conceptual framework encompasses seven dimensions deemed relevant by patients and healthcare providers to offer person-centred care. These dimensions are grouped into three different levels of healthcare delivery: individual level, process level and organizational level.

The seven dimensions of the Patient-centred healthcare delivery framework are presented in Table 1.

**Table 1:** Patient-centred healthcare delivery framework from Mühlbacher et al. 2015

Level	Dimensions	
Individual	1. Interpersonal Care	This dimension includes all elements of the patient-physiotherapist relationship, such as respect, attentiveness, and shared decision-making.
	2. Individualised Healthcare	This dimension involves the use of personalized care interventions based on patient’s needs and context.
Process	3. Coordination and Continuity	This dimension relates to the long-term planning of care, including the collaboration and transitions among and between health care providers and services.
	4. Professional Care	This dimension refers to all aspects of the clinical care, such as patient education, expertise of the healthcare professional and treatment guidelines.
Organizational	5. Data and Information	This dimension refers to the content and the ways in which health information is shared to patients. It also encompasses elements related to the availability, security and transparency of “patient data”, as well as the availability and quality of performance indicators.
	6. Service and Facilities	This dimension refers to the staff within the facilities and to the ‘structural’ aspect of these facilities. More precisely, it includes all aspects related to accessibility and how user-friendly it is, as well as the equipment and furniture inside the facility. The ‘atmosphere’ and ambiance associated within the service are also part of that dimension.
	7. Access	This dimension encompasses both geographical and timely access to services, as well as the costs related to the services.

This analysis was performed by four team members (MAC, NL, GJ, RB) supervised by the first author of the review (JGH). A meeting with the first and the senior authors (JGH and AH) was organized after all the reviewers had each analysed five articles to discuss the analysis process.

### Stage 6: consultation with stakeholders

Our research team included two women living with chronic pain who previously used physiotherapy services (CC, FL). They were recruited from two different associations of persons living with chronic pain in the province of Quebec, Canada. They were both involved in the development of the research protocol for this scoping review, including the elaboration of the specific objectives and research questions. They also participated in the discussions on the analysis process, including the identification and codification of the perceived needs. Their involvement enabled the rest of the team to better understand the reality and lived experience of people living with chronic pain in physiotherapy. Their opinions enriched reflections and analysis of the results, enabling to better identify the perceived needs arising from patients’ experiences, expectations and preferences reported in the included studies.

## Results

**Fig. 1:**
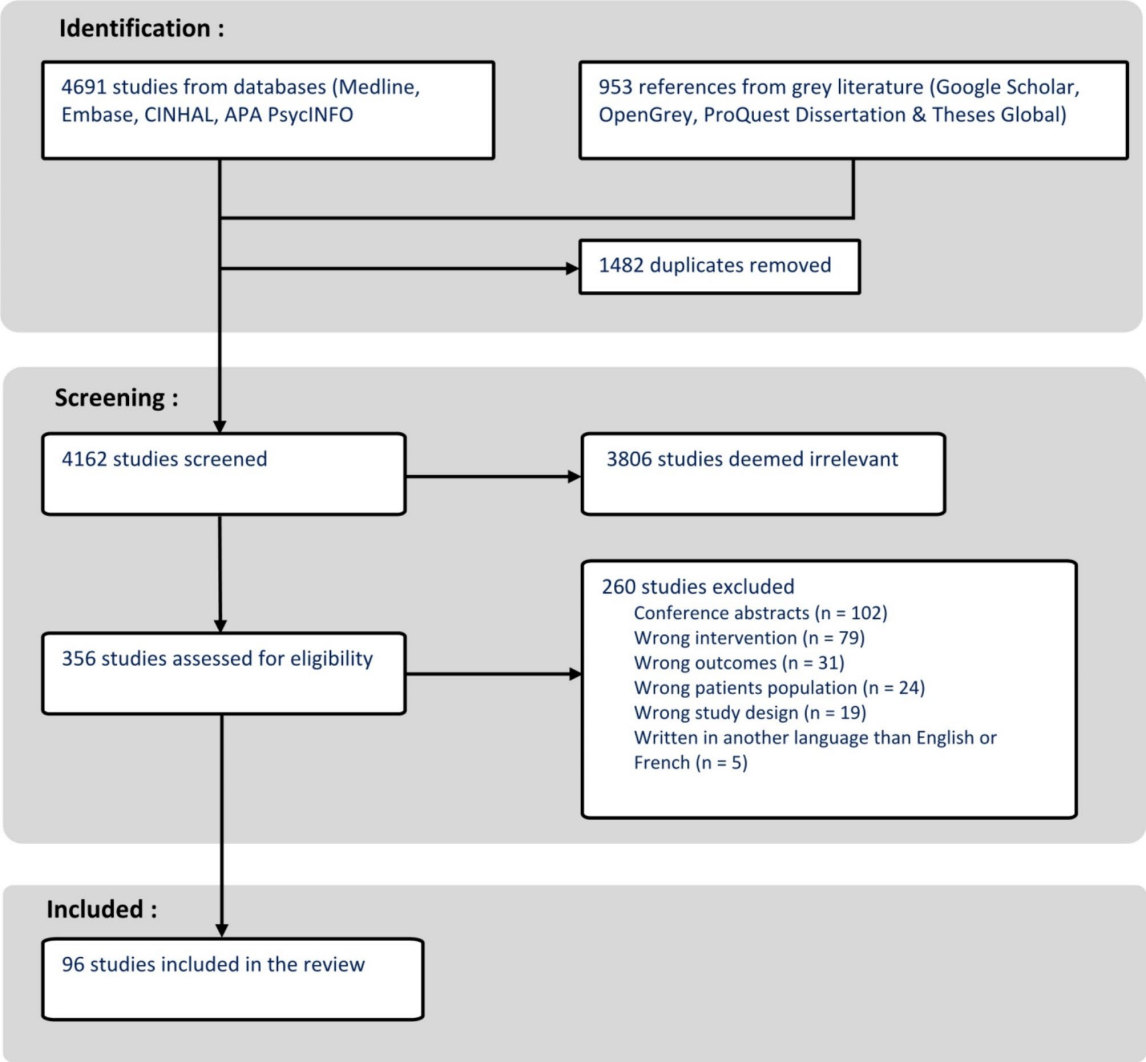
Study selection flow chart

The search in the databases and grey literature resulted in the identification of 5644 studies. After removing duplicates, we screened 4163 studies, of which 96 met the eligibility criteria. These 96 studies where formally included in the final sample and analyzed. Figure 1 shows the detailed study selection flow chart.

### Characteristics of the included studies

Most of the included studies were recently published: 68 of the 96 were published within the last 10 years and only one study was published before 2000. Studies were mainly from Europe (*n* = 62), America (*n* = 17) and Oceania (*n* = 15). Seventy-seven studies used a qualitative design, 7 a quantitative design, 12 a mixed methods design. Although all studies addressed patients’ experiences in physiotherapy and results aligning with our definition of patient’s perceived needs, only five studies specifically mentioned targeting patient’s needs [52–56]. Most studies included participants living with chronic musculoskeletal pain, including all types of arthritis (*n* = 78), but some included participants with a diagnosis of fibromyalgia (*n* = 4), chronic regional pain syndrome (*n* = 1), vulvodynia (*n* = 1), multiple sclerosis (*n* = 1), cancer-related pain (*n* = 1) and spinal cord injury (*n* = 1). Five studies included participants with mixed diagnoses and four studies had participants with chronic pain without any other specific diagnosis. Characteristics of all the included studies are available in Appendix 3.

### Perceived needs of people living with chronic pain regarding physiotherapy services

Our analysis allowed us to identify the perceived needs of people living with chronic pain towards physiotherapy services and to map them to the seven dimensions of the Patient-centred healthcare delivery framework [50]. Most of the codes (perceived needs) resulting from our analysis were mapped into three of those seven dimensions, which are Interpersonal care, Individualised healthcare and Professional care, whereas less codes were mapped into the four other dimensions (Coordination and continuity; Data and information; Service and facilities and Access).

Each of these seven dimensions are presented in the following sections with the perceived needs identified by our inductive coding analysis. These results are summarized in Table 2.

**Table 2:** Seven dimensions of the patient-centred healthcare delivery framework with the corresponding identified perceived needs

Levels of healthcare delivery	Dimensions	Identified perceived needs for/to
Individual Level	Interpersonal Care	• An empathetic relationship [54, 57–83] • Be understood, respected and validated [54, 58, 63, 77, 78, 80, 81, 83–92] • Be considered as full human being in an egalitarian relationship [57, 68, 83, 93–95] • A trusting relationship [56, 57, 61, 64, 68, 70, 72–74, 76, 80, 82, 87, 93, 95–99] • An honest physiotherapist with great communication skills [57, 59, 61, 65, 69, 73, 76, 77, 80, 83, 86, 100] • Support and encouragement [52, 56, 58, 59, 62–64, 66, 67, 69, 74, 77–85, 89, 91–93, 97, 99–112] • Be reassured [52, 56, 57, 61, 67, 69, 72, 76, 77, 84, 85, 87, 97, 100, 106, 110, 113–115] • Collaborate and be actively involved in their rehabilitation [56, 59, 69, 73, 75, 82, 87, 94, 116, 117]
	Individualised Healthcare	• An approach that considers their beliefs, expectations and past experiences in physiotherapy [62, 70, 72, 74, 82, 86, 87, 98, 99, 108, 118–120] • An approach that could help them accept their pain [63, 75, 84, 85, 121] • Autonomy and self-management [52, 61–63, 72, 75, 76, 83–86, 89, 91, 93, 100, 105, 113, 122, 123] • Personalized treatment and objectives [52, 54, 56–59, 63, 65–68, 70, 71, 73–75, 77, 79–85, 87, 89, 91, 92, 94, 97–102, 104, 105, 108–112, 115, 117–120, 122–134] • Social support [62–66, 68, 69, 72, 73, 75, 77, 79–81, 85, 86, 89, 91, 92, 94, 96, 99–101, 104–108, 110–113, 118–120, 135, 136]
Process Level	Coordination and Continuity	• More physiotherapy treatments and follow-up [52, 56, 64–66, 68–70, 72, 77, 80, 84, 87, 92, 99, 111, 118, 121, 123, 126, 130, 135] • Concordant advice and opinion from health professionals [67, 69, 71, 72, 77, 86, 88, 117, 119, 121, 123, 128, 137] • Continuity in physiotherapy and between healthcare professionals [65, 77, 80, 90, 129] • Well-coordinated services [57, 58, 65, 68, 81, 99, 114, 137–140] • Information to be shared between healthcare professionals [69, 70, 97]
	Professional Care	• An expert physiotherapist [54, 56, 57, 63, 65, 70, 72, 73, 76, 78, 82, 84, 86, 87, 91, 105, 109, 112, 114, 123, 125, 126, 139] • A physiotherapist driven by caring intentions [57] • A mindful physiotherapist generous with his time [65, 87, 137, 139] • A proper diagnosis and to know what is “cause of their pain” [52, 56, 58, 61, 67, 70, 81, 85–87, 114, 121, 131, 134, 139] • Education and advice to better understand and manage the pain [52, 53, 56–59, 61–65, 67–73, 75–77, 79–81, 84–87, 89–92, 94, 97–100, 102, 110–112, 114, 115, 117, 119–123, 126, 127, 130, 133, 135–138, 141, 142] • Information on physiotherapy treatments and its possible outcomes [55, 59, 65, 67, 70, 81, 82, 87, 88, 97, 103, 112, 114, 119, 121–123, 126, 128, 143] • A clear, adapted and supervised exercise program [52, 53, 56, 59, 63, 64, 67, 73–77, 79–82, 84, 85, 87, 91, 92, 97–111, 116, 119, 121, 123–125, 127, 129–135, 142, 143] • Regular feedback [71, 72, 74, 98, 102, 109, 110, 120, 125] • Passive intervention such as manual therapy or other analgesic modalities [60, 63, 67, 72, 82, 87, 88, 115, 123, 129, 130, 134, 141, 143] • Information and advice related to psychosocial health [62, 72, 119, 120, 129, 142] • Pain relief and sustainable outcomes from physiotherapy interventions [58, 62–64, 67, 68, 70, 72, 74, 76, 77, 79, 80, 82, 85, 86, 88, 90, 93, 96, 99–105, 107, 110, 111, 114, 115, 118, 120, 134, 135, 137, 138]
Organizational Level	Data and Information	• Information on direct access to physiotherapy and reimbursement opportunities [97, 143] • Information on complementary services and on sensitive topics [53, 62, 75, 110, 133]
	Services and Facilities	• Simple, convenient and efficient in-clinic services [52, 57, 65, 68, 69, 74, 76, 77, 82, 84, 90, 92, 93, 100, 102, 105, 110, 112, 125, 135] • Easy to use telerehabilitation services and adequate home installations [74, 88, 93, 97, 100–102, 105, 110, 125, 134, 135, 139, 144–146] • Face-to-face appointments [52, 93, 112, 131, 134, 144, 146] • Complementary services and infrastructures [52, 54, 62, 79, 80, 92, 104, 107, 108, 110, 112, 133, 138]
	Access	• Easy access to nearby physiotherapy services [58, 62, 63, 74, 77, 82, 92, 93, 104, 110, 123, 137–140, 147] • Quick and flexible appointments [52, 58, 62, 65, 68–70, 76, 82, 90, 99, 110, 112, 121, 133, 137, 140] • Low-cost physiotherapy services [54, 55, 58, 69, 72, 74, 80, 82, 88, 97, 100, 103, 111, 112, 117, 122, 123, 130, 134, 135, 137–140, 143, 144] • Transportation, accommodation and being accompanied [68, 79, 137, 140, 144]

### Dimension 1: interpersonal care (individual level)

This dimension relates to elements of the patient-physiotherapist relationship. Sixty-four of the 96 studies reported patients’ perceived needs associated with this dimension of the framework.

One of the frequently discussed needs was the need for participants to establish an empathetic relationship with their physiotherapist [54, 57–83]. Many participants expressed the need to be understood, respected and validated by their therapists throughout their rehabilitation [54, 58, 63, 77, 78, 80, 81, 83–86]. Regardless of their age [87], their condition [83, 88–90], their life experience [81, 84, 91, 92] or their choice of medication [81], they wanted to be respected and recognized as full human beings [83, 93] within an egalitarian relationship [57, 68, 93–95]. Another often evoked need related to the patient-physiotherapist relationship was the need for a therapeutic relationship based on trust [56, 57, 61, 64, 68, 70, 72–74, 76, 80, 82, 88, 93, 94, 96–99]. To foster such an empathetic and trusting relationship, many studies reported the need for the participants to have an honest physiotherapist [57, 73, 76] with great communication skills [59, 61, 65, 69, 73, 76, 77, 80, 83, 86, 100].

The needs to be supported and encouraged [52, 56, 58, 59, 62–64, 66, 67, 69, 74, 77–85, 89, 90, 92, 94, 97, 99–112] and to be reassured by their physiotherapists [52, 56, 57, 61, 67, 69, 72, 76, 77, 84, 85, 88, 97, 100, 106, 110, 113–115] were also commonly found in the included studies. Being reassured helped some participants to better manage their exercises in relation to pain, i.e. to know when to stop or progress their exercises [77], to diminish their fear and to increase their motivation [85, 97, 110].

Finally, in many studies, participants highlighted the need to collaborate and be actively involved in their rehabilitation [56, 59, 69, 73, 75, 82, 88, 95, 116]. Participants were willing to collaborate if they had treatment options to choose from and if their opinion was truly considered by the physiotherapist [56, 117].

### Dimension 2: individualized healthcare (individual level)

This dimension includes the personalization of care to respect patients’ context and answer their needs. Seventy-eight studies reported needs related to this dimension.

Many studies highlighted the need for participants to have a physiotherapist that considered their beliefs, expectations and past experiences, as these elements could influence their experience in physiotherapy [62, 70, 72, 74, 82, 86, 88, 98, 99, 108, 118–120]. Some participants also expressed the need to accept their pain and how physiotherapy interventions could help them in this regard [63, 75, 84, 85, 121].

Various studies reported a perceived need for participants to be given or informed of strategies and advice to enhance the self-management of their condition [52, 61–63, 72, 75, 76, 83–86, 89, 92, 94, 100, 105, 113, 122, 123]. Participants in the studies sought empowerment [57, 76, 124], independence [57, 63, 66, 106] self-investment [63, 70, 99, 100, 118, 120], self-accomplishment [102, 104], self-consciousness [84, 86, 93, 96, 109] and control over their life and condition [57, 63, 86, 105, 109]. All these elements contributed to enhance their need for autonomy and self-management.

Moreover, several included studies reported that participants wished to feel unique [54, 57, 58, 66, 69, 71, 74, 100, 125], which made them search for personalized treatments. Indeed, many participants reported that they did not want a one-size-fits-all approach to care [56, 66, 68, 70, 73, 80, 82, 83, 85, 109, 126]. According to participants in several studies, physiotherapy services needed to be adapted to their physical capabilities [57, 65, 68, 71, 74, 111]; their language [57, 97]; their literacy level [100]; their culture [57]; their financial constraints [52]; their employment status [112]; their busy schedule [77, 90, 101, 102, 105, 110, 112, 113, 122]; and their levels of energy [67, 75, 112, 113, 115, 127]. Other participants wanted the length and frequency of the sessions adapted to their preferences [109, 128]. Some studies also discussed the need for participants to be involved throughout the whole process of treatment planning and objectives setting [56, 63, 82, 88, 95, 117, 129]. Few participants specifically asked for meaningful objectives [66, 81, 119], both short and long-term [80], that respected their preferences and limits [57, 68].

In a large amount of studies, participants reported the need to receive personalized exercises [52, 54, 58, 59, 63, 65, 66, 71, 74, 77, 80, 81, 83–85, 88, 89, 92, 95, 97–100, 102, 104, 105, 108–112, 115, 117, 118, 120, 123, 124, 127, 128, 130–135]. More precisely, it was important for the participants to have exercises adapted to their lifestyle [59, 74, 80, 85, 97, 108, 123, 132] and easy to integrate in their daily routine [52, 65, 71, 74, 80, 99, 100, 110, 111, 115, 117, 120, 127]. The complexity [63, 74, 100, 102, 110, 127], pace [95, 105], quantity [110, 124, 134], duration [97, 110, 123, 124], amount of required supervision [80] and level of difficulty [77, 80, 84, 92, 110] were among the most important aspects that needed personalization for the participants. Some also needed exercises that were well-adapted to the variability of their pain [68, 79, 108, 111, 124, 130, 131] and that did not increase their pain [68, 77, 84, 98, 99, 110].

Several studies mentioned the need for participants to be supported by their close ones [63, 69, 72, 75, 77, 80, 81, 86, 89, 100, 105, 108, 110–112, 118] and to participate in exercise and support groups [62, 64–66, 68, 69, 72, 73, 77, 79–81, 85, 89, 90, 92, 95, 96, 99–101, 104–108, 110–113, 119, 120, 136, 137]. Among other things, being in a group was seen as a great source of motivation [62, 64, 66, 69, 80, 81, 92, 96, 104, 105], and allowed them to feel understood and accepted by others [66, 69, 73, 89, 92, 99, 107, 136]. However, some participants preferred individual sessions [65, 79, 80, 112, 136].

### Dimension 3: coordination and continuity (process level)

This dimension involves elements related to the long-term planning of care and the collaboration between healthcare professionals. Twenty-nine of the 96 included studies reported perceived needs associated with it.

Several studies highlighted the need for participants to receive more physiotherapy treatments (more frequently, longer sessions and for a longer term) [65, 68–70, 72, 77, 84, 88, 111, 118, 121, 128, 132]. Some participants expressed the need to have a follow-up with their physiotherapist after finishing their episode of care [52, 56, 64, 66, 69, 80, 90, 99, 123, 136].

In numerous studies, participants mentioned their need to receive concordant advice and opinion from the various health professionals consulted [67, 69, 71, 72, 77, 86, 87, 117, 119, 121, 123, 125, 130]. Some participants also perceived a need for continuity among the physiotherapists they consulted, as they wanted to be able to see the same therapist between treatments or episodes [65, 77, 80, 91, 131]. Another frequently reported need was for well-coordinated services, especially to be referred to the right professional at the right time [57, 58, 68, 81, 99, 114, 138]. When possible, patients also liked to see all their healthcare professionals on the same day [68, 125, 139, 140].

In two studies, some participants mentioned the need for the physiotherapists working within the service to have good communication between them and with the administrative staff to ensure the appointments are well organised [65, 139].

Some participants also perceived a need for information related to their medical history, their reasons for consulting in physiotherapy and their medical imagery results between healthcare professionals [69, 70, 97].

### Dimension 4: professional care (process level)

This dimension includes all aspects of clinical care such as patient education, expertise of the physiotherapist and interventions used. This dimension was the most discussed among the seven dimensions of the framework, as 87 of the studies presented needs associated with professional care.

A great number of studies reported that participants wished to be treated by an expert and competent physiotherapist [54, 56, 57, 63, 65, 70, 72, 73, 76, 78, 82, 84, 86, 88, 92, 105, 109, 112, 114, 123, 127, 128, 139]. As some studies mentioned, this expertise increases participants’ confidence [65, 76, 78, 112] and gives them a feeling of safety as they feel they are being treated by someone who truly knows what he or she is doing [70, 73, 76, 92, 109, 112].

One study mentioned the importance for participants to have a physiotherapist that is driven by caring intentions, and not by primarily making money or fulfilling requirements for a third party [57]. In addition, few studies raised participants’ need for a mindful therapist [65] that is generous of his or her time to decently answer their questions [88, 125, 139].

Some participants also expressed the need to receive a proper diagnosis [58, 67, 70, 81, 85, 121, 135, 139] and to know what is the “cause of their pain” [56, 61, 88, 98, 133]. This search for a diagnosis also led some participants to perceive the need to undergo medical imagery to be able to “see” their condition and what is problematic in their body [52, 70, 81, 86, 114].

Numerous studies showed that participants needed education [52, 53, 56–59, 61–65, 67, 69–73, 76, 77, 79–81, 84, 85, 89–91, 95, 97–100, 102, 110, 112, 114, 115, 119, 121–123, 125, 128, 129, 132, 137, 138, 141] as well as advice to better understand and manage their pain [52, 58, 61, 65, 67–69, 72, 75, 76, 80, 84–86, 88–92, 95, 100, 110–112, 114, 115, 117, 120, 121, 132, 134, 136, 141, 142]. In several studies, participants also said they wished to receive explanations regarding their treatments and their possible outcomes [55, 59, 65, 67, 70, 81, 87, 88, 97, 112, 114, 119, 121, 123, 128, 130, 143]. Some participants also said they wanted to better understand how physiotherapists can help them compared to other healthcare professionals [82, 103, 122].

The need for participants to have an exercise program was frequently mentioned in the included studies [52, 56, 59, 63, 67, 73, 75, 77, 80–82, 84, 85, 88, 92, 97, 100, 104, 106, 110, 116, 121, 126, 131–133, 135, 142, 143]. When taught the exercises, participants said they liked clear instructions supported by physiotherapist’s demonstrations [59, 63, 64, 67, 74, 76, 77, 80, 84, 89, 90, 100, 103, 107, 110, 123, 124, 127, 133, 134, 136]. They also needed to be supervised [73, 79, 80, 85, 92, 101, 102, 105, 109–111, 119, 124, 127, 134, 136]. As mentioned in some studies, participants needed to receive regular feedback while performing the exercises [71, 74, 102, 109, 110, 120, 127] or to have access to tools to monitor themselves and provide them with feedback, without the physiotherapist being present [72, 98]. Other participants also needed a program to help them to progressively return to their activities, especially if it allowed them to reach short-term goals and to gradually progress their skills [79, 80, 99, 119, 121, 129, 133]. In many studies, participants wanted the exercises to be adapted to their pain level [80, 97, 98, 100, 108, 110, 111, 121, 127, 131, 143].

On the other hand, several participants of the studies mentioned the need to receive passive interventions such as manual therapy [63, 67, 72, 88, 115, 123, 131, 132, 135, 141, 143] or other analgesic modalities like transcutaneous electrical nerve stimulation (TENS) [87], taping [82] and subdermal needling [60].

Other than needs related to their physical wellbeing, some of the studies highlighted the need for participants to receive information and advice regarding their psychological health [72, 119, 131]. To that extend, other participants expressed the need to learn more about mindfulness and/or emotional management techniques to help them accept and live with their pain condition [62, 72, 120, 142].

Finally, several studies highlighted the need for participants to experience pain relief with physiotherapy sessions [58, 62, 67, 68, 70, 72, 74, 76, 79, 80, 82, 85–87, 91, 94, 99–104, 110, 114, 115, 118, 120, 125, 135, 136, 138] or a halt in the deterioration of symptoms [63, 77]. In addition to pain relief, depending on their limitations and conditions, some participants mentioned that they needed to see improvements regarding their balance [63, 96], muscle strength [74, 82, 104, 105, 107], mobility [70, 96, 104, 111], cardiovascular endurance [120] and well-being [64, 87], as well as a decrease in their fear of movement [101]. Improved quality of life, function and return to meaningful activities were also described as desirable outcomes of physiotherapy treatment [94, 102, 111].

### Dimension 5: data and information (organizational level)

This dimension refers to the content and the way in which health information is shared to patients. Seven of the included studies addressed perceived needs related to this dimension.

In one study, participants mentioned the need to be informed of direct access in physiotherapy, i.e. access without a medical reference [143], whereas another study reported the need of participants to receive information on reimbursement opportunities for healthcare services such as physiotherapy [97]. In few other included studies, participants expressed the needs to receive information on complementary services, such as aqua gym [62, 134] and on health via online resources such as websites [110] or books [75]. As for sensitive topics such as sexuality, some participants wanted to have access to information to read or watch, such as pamphlets, rather than discussing it directly with their physiotherapist [53].

### Dimension 6: services and facilities (organizational level)

This dimension of the Patient-centred healthcare delivery framework includes the structural (physical) aspects of the facilities, the user-friendliness of the services and the helpfulness of the employees. Thirty-eight of the included studies mentioned needs associated with this dimension.

Several of the included studies reported that participants needed simple, convenient and efficient clinic services [57, 65, 68, 69, 76, 82, 84, 91, 94, 105, 110, 136]. Participants wanted to be able to book their appointments online [69, 76, 110] and to be able to easily modify an appointment [65]. Some also appreciated being able to access several healthcare services at the same place [82] and to discuss with the clinic’s administrative staff in order to rapidly book an appointment in the case of an urgent and/or acute situation [69, 110]. To help them remember their appointments, some participants mentioned they wanted to receive reminders [90, 100, 102, 105, 110, 127]. Participants of a few studies also wanted to be able to use phone calls [52, 74, 77, 110, 112] or to send text messaging [77] or emails [77] to reach their physiotherapists when needed. Once arriving at the facility, some participants mentioned that they needed to have easy physical access to the facility where physiotherapy was provided with parking spots [68, 94, 136] or bus stops [68] available close by. Inside the facility, some participants mentioned they appreciated a clean and calm environment [57], with enough space or installations to do their exercises [84, 105], and that provided enough individual resources such as bedsheets to ensure a clean and healthy environment [91].

Several studies reported the need for participants to receive telerehabilitation physiotherapy services [74, 87, 94, 97, 100–102, 105, 110, 127, 135, 136, 139, 144–146], although some preferred to have face-to-face appointments [52, 94, 112, 133, 135, 144, 146]. To take full advantage of telerehabilitation, participants raised the need to have an adequate internet access [94, 135, 146] and a camera [74, 135], as well as to be comfortable using these technologies [94, 101]. They also needed to possess enough space and equipment to properly perform their exercises [87, 97, 100, 102, 105, 110, 127, 135, 136, 146].

To complete prescribed activities or exercises, people living with chronic pain expressed the need to have access to facilities such as gyms, recreational centers and swimming pools [52, 54, 62, 79, 80, 90, 104, 107, 108, 112, 134, 138]. As some gyms could feel intimidating [80], some participants said they could initially need support to get familiarized and increase their confidence to access these facilities [112]. Finally, when using these facilities, participants also perceived the need to be supervised by well trained professionals to select and tailor the exercises to their condition [110, 134].

### Dimension 7: access (organizational level)

This last dimension relates to geographical and timely access as well as costs related to the services offered. Forty-four of the included studies reported perceived needs related to access to physiotherapy services.

Many studies mentioned the need for participants to access services close to their home [58, 62, 74, 77, 82, 90, 94, 104, 125, 138–140], to receive home-based physiotherapy services [63, 77, 110, 147] or to have access to outpatient physiotherapy services at the hospital [123]. In addition, some participants also needed to have quick access for physiotherapy services, as they frequently said that they faced long delays and waiting lists to obtain such services [58, 62, 65, 68–70, 110, 121, 125, 140]. Others wanted to get access to physiotherapy services when needed, without the need to consult a doctor first for a reference [52, 76]. Many participants also expressed the need for allowing flexibility in scheduling appointments to better accommodate their lifestyle [62, 69, 70, 76, 82, 91, 99, 112, 134, 140].

Participants of several of the included studies perceived a need for affordable services [54, 58, 72, 82, 100, 103, 111, 112, 117, 122, 125, 135, 136, 138, 140, 144] or services covered by insurances [55, 58, 69, 72, 80, 87, 97, 123, 132, 138, 143]. In this way, some participants expressed the need to reduce the indirect costs related to their physiotherapy appointment, such as a drop in income due to repetitive absences from work [80, 112, 138, 140], babysitting costs [111, 112, 135] or costs related to transportation [74, 125, 135, 139, 140, 144]. To this end, some participants perceived a need for transportation such as public transport, adapted transport (i.e. paratransit) or transport provided by community services to get to their appointment [79, 125, 140, 144]. Others also said they needed to be accompanied to travel to their physiotherapy treatments [68, 79, 125, 140] or to have accommodation because the physiotherapy services were situated too far from their homes, which meant spending a night away [140].

## Discussion

To explore the perceived needs of people living with chronic pain towards physiotherapy services, we conducted a scoping review of 96 studies. Most of them were studies of qualitative design and included participants living with chronic musculoskeletal pain.

The use of the patient-centred framework to map the perceived needs identified showed that most studies reported needs associated with the dimensions of interpersonal care and individualized healthcare (both at the Individual Level), as well as professional care (Process Level). Studies mentioning needs related to the dimensions of access (Organizational Level); coordination and continuity (Process Level); or services and facilities (Organizational Level) were less frequent, while very few studies mentioned needs associated with the dimensions of data and information (Organizational Level).

The interpersonal aspect of the care relationship was commonly present in the studies we analyzed. Our results show that participants perceived important needs associated with respect, understanding, empathy, honesty and communication skills of physiotherapists. These results are closely linked to the qualities of a “good” physiotherapist recently identified by Kleiner et al. in an integrative review of 27 qualitative studies [148]. According to their results, patients and physiotherapists consider a “good” physiotherapist to be responsive, ethical, communicative, caring, competent and collaborative [148]. For example, according to the authors, participants defined a “good” physiotherapist as someone who is attentive, a good listener, reassuring, understanding, empathetic, humble, honest and respectful. A “good” physiotherapist must also communicate clearly, foster collaboration and have the appropriate knowledge and practical skills. The authors concluded that “a ‘good’ physiotherapist balances technical competence with a human way of being when interacting with patients” (p.107) [148]. This balance between technical and interpersonal skills is also at the heart of the consensus-based competency profile for pain management recently proposed by a Delphi study involving representatives from Canadian university physiotherapy programs, clinical educators and individuals living with chronic pain [149]. This interesting conclusion shows that patients’ perceived needs toward physiotherapy services and the qualities of a “good” physiotherapist are well-aligned, as the perceived needs identified in our review were mainly mapped into the dimensions of “interpersonal care” and “professional care”.

For people living with chronic pain, the need for a healthy therapeutic relationship, based on listening and empathy, may stem from a perceived lack of understanding on the part of those around them. Indeed, often feeling stigmatized by family, friends and even by some healthcare professionals, people living with chronic pain might seek recognition and empathy from their physiotherapist [25, 81, 150].

Furthermore, many of the perceived needs identified in our review are in line with the findings of recent reviews on patients’ perceptions of healthcare services. In a 2020 overview of reviews on patients’ perceptions regarding their experiences with healthcare services, Chi-Lun-Chiao et al. identified the elements deemed important for people with musculoskeletal disorders [151]. Similar to our results, the authors underlined the importance of communication and interpersonal relationships as patients desired a physiotherapist who understood and respected them and communicated clearly. They also mentioned patients’ expectations to be educated about their condition, to be involved in decision-making surrounding their rehabilitation, and to receive individualized interventions. The participants in the analyzed reviews also considered as important the competencies and technical skills of healthcare providers. Finally, the authors reported various patients’ preferences with regards to the organization of healthcare. For example, they mentioned the need for continuity and coordination of care, as well as the need for access to care and to have flexible appointment times. Although the results of this study and our own show these many similarities, Chi-Lun-Chiao et al. reported elements associated with receiving care in a safe environment and issues about the complexity of paperwork related to healthcare, which were not raised by our review.

Perceived needs related to organizational aspects were less addressed in the studies included in our review. Although some of the studies we analyzed mentioned perceived needs related to access to physiotherapy, we might have expected more studies on the subject, given that unmet needs related to access to healthcare services for people living with chronic pain (including access to physiotherapy) are often discussed in the literature, especially from an experts’ evaluation perspective [152–156]. It is also surprising that few of the included studies mentioned needs related to the physical environment given that several published studies have discussed how such organizational factors can influence patients’ experiences in healthcare [157–163]. Furthermore, some of these organizational factors are directly related to certain perceived needs identified in our review, such as needs concerning communication and interpersonal relationships. In a 2016 systematic review on factors perceived by patients and physiotherapists as influencing their interactions, O’Keeffe et al. [162] mentioned the importance of appointment length and flexibility when booking appointments. Patients appreciated having more time with therapists to talk and were grateful when therapists could adjust the timing of their appointments to suit their needs [162]. In another study, Morera-Balaguer et al. also mentioned how organizational factors such as the lack of coordination between healthcare professionals and a privacy-free environment could affect the therapeutic relationship in physiotherapy [163]. The impact on teamwork and communication of the design of medical facilities was also addressed in Gharaveis et al.’s 2018 systematic review [159]. The results of this review emphasized the impact of aspects of environmental design, such as spatial layout, furnishing, size, space and privacy of rooms, on physicians and nurses’ interactions, as well as communication with patients. The authors also mentioned how room lighting influences interactions in healthcare facilities, as dim lighting resulted in a sensation of calmness for patients and helped facilitate longer conversations. More recently, Zaniboni et al. studied the lighting conditions in physiotherapy facilities and showed that natural day light was associated with higher satisfaction from patients and therapists [160].

Beyond the influence of the environmental design on satisfaction and interactions in healthcare facilities, the environment of physiotherapy services could have some insidious and negative influence on patients’ experiences in physiotherapy. Indeed, some studies explored the influence of physiotherapy services physical environment on power relations between physiotherapists and patients [164, 165]. The authors of these studies argue that the medical look of the décor (e.g. white walls with anatomical posters) and the equipment present in physiotherapy clinics (particularly the treatment bed) suggest a biomedical approach of physiotherapy where the person is reduced to a body and the physiotherapist takes on an expert posture. Thus, as soon as the person enters the waiting room of the physiotherapy clinic, he or she transitions into a potentially “inferior” posture compared to the expert physiotherapist [164]. Such a power imbalance can affect the relationship between physiotherapists and patients, and hinder their collaboration [166]. It might thus be more uncomfortable for patients to express their preferences regarding the organizational elements of healthcare. They may also not see the links between these elements and their lived experience during care. These reasons could potentially explain the lack of perceived needs related to the organizational aspects of physiotherapy services found in the articles included in our review. It is thus essential that more research explores and considers the perceived needs of patients in relation to organizational elements such as environmental design.

Other than the perceived needs identified by our review, an interesting finding is that although meeting patients’ needs are regularly put forward by researchers and healthcare experts, only five of the 96 included studies directly named addressing people’s needs. That is, only five studies explicitly used the term “needs” in the study title or objectives. Although all the other studies revealed elements that enabled us to identify perceived needs according to the definition of such needs retained for the review, these studies used terms such as expectations, preferences, and so on. Moreover, the only study that directly addressed patients’ needs did not provide a definition of what a need is. This may not be surprising because a need is complex and ill-defined.

This finding reinforces existing questions about the use and distinctions between the terms “needs” and “expectations” or “desire”. For some authors, expectations (or wants/demands/desire) and needs should be considered as two different constructs [15, 167, 168]. Expectations seemed more accepted to talk about a person’s demand or desires [168], whereas a need was often linked to a moral obligation to do everything possible to meet it [169]. Therefore, making a distinction between an expectation and a need based on someone’s demand could serve as justifications for physiotherapists and health professionals to attribute less importance to a person’s demand, framing it as a preference or an expectation of that person, and not a need that should be met. However, as Brock [169] illustrated when talking about Internet access, the distinction between a need and a desire is sometimes very thin and can evolve over time. Initially perceived as a desire, the Internet is now more widely accepted in society than ever before and thus, recognized as a need.

Nonetheless, to avoid any confusion or temptation to distinguish an expectation from a need, or more precisely from a perceived need, it is crucial to pursue efforts to more clearly define “perceived needs”. In this respect, Schweighart et al. [170] have recently proposed a definition of “needs and wishes” that could inform what a perceived need should be: “Any desire or craving that the person subjectively feels within him- or herself, whether this is material or immaterial, for change or preservation, already fulfilled or still unfulfilled, realistic or unrealistic, current or future, more or less urgent. The fulfillment of this desire causes a positive effect within the person. This positive effect can be related to the quality of life, satisfaction, self-image, autonomy, and any other aspect of the person’s life.” (p.2). Recognizing that a person’s demands, preferences and expectations can all be defined as a perceived need is certainly a key element for physiotherapists to better recognize and respond to their patients’ needs.

### Strengths and limitations

One of the potential challenges of this study concerns the inclusion criteria and keywords used in our research strategy surrounding perceived needs. Since there is no clear and universally accepted definition of the term “perceived needs”, we decided to include interrelated constructs such as demands, preferences and expectations, as described previously. Although this methodological choice may be seen as a limitation of our study, we also believe it can be seen as a strength. Indeed, by including articles dealing with demands, preferences, expectations and lived experiences, we gathered a large sample of studies, the vast majority of which would not have been included if we had only used the keyword “perceived needs”. To this end, we created a comprehensive research strategy with the help of two librarians from two different universities. However, by limiting our research to physiotherapy services, it is likely that some perceived needs, possibly applicable to physiotherapy services but presented in the literature as related to other health services for persons experiencing chronic pain, may have been missed. For example, few needs associated with the multidisciplinary aspect of care were identified by our analysis, even though it is promoted as a key element in rehabilitation for people living with chronic pain [171, 172].

Methodological quality or risk of bias of the studies included was not assessed in our review. This decision aligns with the recommendations for conducting a scoping review [44, 173, 174] and was taken in the light of the fact that our review aims to draw up an exhaustive list of the needs perceived in the literature in order to understand their scope. Consequently, while some perceived needs identified in our review are more frequent than others in the literature, our review does not enable us to draw conclusions about the relative importance of any specific needs.

This review aimed to identify and assess the extent of the perceived needs related to chronic pain, without focusing on specific types of chronic pain, physiotherapy interventions, or geographic regions. As a result, no comparative analysis was conducted.

The active involvement of two patient-partners throughout this review is also a strength of our study. Their input during the analysis process enabled us to ensure the completeness and accuracy of the needs identified and formulated, often based on patients’ preferences or lived experiences described in the included studies. All methodological decisions made during this research were also recorded in a logbook to ensure our entire methodological process was rigorous and transparent.

## Conclusion

A better understanding of the perceived needs of people living with chronic pain in regards of physiotherapy services is a key step towards better considering and respecting these needs in physiotherapy. The findings of this review highlight the diverse and heterogeneous nature of perceived needs. Indeed, the perceived needs identified through the scoping review were mapped within the seven dimensions of the Patient-centred healthcare delivery framework. Future research is also needed to obtain a consensus definition of a perceived need, and to explore in more detail patients’ perceived needs with regards to organizational elements of care, such as the physical environment. Finally, although our review targeted the perceived needs related to physiotherapy services for people living with chronic pain, it is likely that some of our findings are relevant to other healthcare services or conditions. Indeed, beyond being treated by a knowledgeable person, what patients say they need is to feel respected, understood and included as full human beings throughout their care.

## Electronic supplementary material

Below is the link to the electronic supplementary material.

Supplementary Material 1.

Supplementary Material 2.

Supplementary Material 3.

## Data Availability

No datasets were generated or analysed during the current study.
