# Supplementary material for: “How can we help you?”: results of a scoping review on the perceived needs of people living with chronic pain regarding physiotherapy
Source: BMC Health Serv Res. 2024 Nov 14;24:1401. doi: 10.1186/s12913-024-11805-3 (PMC11562623; doi:10.1186/s12913-024-11805-3)
Supplement: Supplementary file 2 — Supplementary Material 2. [file 12913_2024_11805_MOESM2_ESM.docx]

## Appendix 2 – Search strategies in Medline, PsycInfo, Embase and CINHAL.

**Search Strategy: Medline-Ovid**

| **Perceived needs** | **Chronic pain** | **Physiotherapy services** |
| --- | --- | --- |
| Needs Assessment/    Health Services Needs and Demand/  ((unmet or met or perceived or felt or expressed or subjective or healthcare) adj3 needs).ab,ti.  ((patient* or people or client* or person* or user*) adj3 (need* or expect* or experience* or perception* or satisf* or perspective* or desire* or prefer* or belief* or view* or want* or attitude*)).ab,ti.  Combine using ‘OR’ | Chronic Pain/  Fibromyalgia/  Complex Regional Pain Syndromes/  exp Arthritis/  (((Chronic or persistent) adj3 (pain or condition*)) or fibromyalgia or “complex regional pain syndrome” or arthritis).ab,ti.  Combine using ‘OR’ | Physical Therapy Modalities/  Physical Therapists/  Musculoskeletal Manipulations/  Exercise Therapy/    (physiotherap* or “physical therap*” or “manual therap*” or kinesiotherap*or “musculoskeletal manip*”).ab,ti.    Combine using ‘OR’ |
| Combine using ‘AND’ | | |

**Search Strategy: APA PsycInfo**

| **Perceived needs** | **Chronic pain** | **Physiotherapy services** |
| --- | --- | --- |
| Needs/  Health Service Needs/  Needs Assessment/  need satisfaction/  ((unmet or met or perceived or felt or expressed or subjective or healthcare) adj3 needs).ab,ti.  ((patient* or people or client* or person* or user*) adj3 (need* or expect* or experience* or perception* or satisf* or perspective* or desire* or prefer* or belief* or view* or want* or attitude*)).ab,ti.  Combine using ‘OR’ | chronic pain/  complex regional pain syndrome (type i)/  fibromyalgia/  exp arthritis/  (((Chronic or persistent) adj3 (pain or condition*)) or fibromyalgia or "complex regional pain syndrome" or arthritis).ab,ti.  Combine using ‘OR’ | physical therapy/  physical therapists/  (physiotherap* or “physical therap*” or “manual therap*” or kinesiotherap*or “musculoskeletal manip*”).ab,ti.    Combine using ‘OR’ |
| Combine using ‘AND’ | | |

**Search Strategy: Embase**

| **Perceived needs** | **Chronic pain** | **Physiotherapy services** |
| --- | --- | --- |
| needs assessment/  health care need/  ((unmet or met or perceived or felt or expressed or subjective or healthcare) adj3 needs).ab,ti.  ((patient* or people or client* or person* or user*) adj3 (need* or expect* or experience* or perception* or satisf* or perspective* or desire* or prefer* or belief* or view* or want* or attitude*)).ab,ti.  Combine using ‘OR’ | chronic pain/  exp complex regional pain syndrome/  fibromyalgia/  exp arthritis/  (((Chronic or persistent) adj3 (pain or condition*)) or fibromyalgia or "complex regional pain syndrome" or arthritis).ab,ti.  Combine using ‘OR’ | physiotherapy/  home physiotherapy/  joint mobilization/  physiotherapy practice/  kinesiotherapy/  musculoskeletal manipulation/  spine manipulation/  (physiotherap* or "physical therap*" or "manual therap*" or "kinesiotherap*or musculoskeletal manip*").ab,ti.    Combine using ‘OR’ |
| Combine using ‘AND’ | | |

**Search Strategy: CINHAL**

| **Perceived needs** | **Chronic pain** | **Physiotherapy services** |
| --- | --- | --- |
| MH "Health Services Needs and Demand"  MH "Needs Assessment"  TI ( ((unmet or met or perceived or felt or expressed or subjective or healthcare) N3 needs) )  AB ( ((unmet or met or perceived or felt or expressed or subjective or healthcare) N3 needs) )  TI ( ((patient* or people or client* or person* or user*) N3 (need* or expect* or experience* or perception* or satisf* or perspective* or desire* or prefer* or belief* or view* or want* or attitude*)) )  AB ( ((patient* or people or client* or person* or user*) N3 (need* or expect* or experience* or perception* or satisf* or perspective* or desire* or prefer* or belief* or view* or want* or attitude*)) )  Combine using ‘OR’ | MH "Chronic Pain"  MH "Fibromyalgia"  MH "Complex Regional Pain  Syndromes"  MH "Arthritis+"  TI ( ((Chronic or persistent) adj3 (pain or condition*)) or fibromyalgia or "complex regional pain syndrome" or arthritis) ) )  AB ( ((Chronic or persistent) adj3 (pain or condition*)) or fibromyalgia or "complex regional pain syndrome" or arthritis) ) )  Combine using ‘OR’ | MH "Physical Therapy Practice"    MH "Home Physical Therapy"  MH "Physical Therapy Service"  MH "Physical Therapist Assistants"  MH "American Physical Therapy Association"  MH "Physical Therapy Assessment"  MH "Physical Therapy"  MH "World Confederation for  Physical Therapy"  MH "Canadian Physiotherapy Association"  MH "Manual Therapy"  MH "Therapeutic Exercise"  TI ( (physiotherap* or "physical therap*" or "manual therap*" or kinesiotherap*or "musculoskeletal manip*") )  ( (physiotherap* or “physical therap*” or “manual therap*” or kinesiotherap*or “musculoskeletal manip*”) )  Combine using ‘OR’ |
| Combine using ‘AND’ | | |
