# Supplementary material for: “How can we help you?”: results of a scoping review on the perceived needs of people living with chronic pain regarding physiotherapy
Source: BMC Health Serv Res. 2024 Nov 14;24:1401. doi: 10.1186/s12913-024-11805-3 (PMC11562623; doi:10.1186/s12913-024-11805-3)
Supplement: Supplementary file 3 — Supplementary Material 3. [file 12913_2024_11805_MOESM3_ESM.docx]

## Appendix 3 - Characteristics of the included studies

| **Authors** | **Publication date** | **Title** | **Location** | **Study objectives** | **Study design and data collection methods** | **Type of chronic pain** |
| --- | --- | --- | --- | --- | --- | --- |
| Ackerman et al.(136) | 2015 | Personal Perspectives on Enablers and Barriers to Accessing Care for Hip and Knee Osteoarthritis | Australia | "[…] to explore perceived factors affecting access to conservative and surgical treatment for hip and knee OA." (p.27) | Qualitative - semi structured interviews | Hip and knee osteoarthritis |
| Antcliff et al.(125) | 2021 | “Pacing does help you get your life back”: The acceptability of a newly developed activity pacing framework for chronic pain/fatigue | England | "[…] to explore the acceptability of using the newly developed activity pacing framework in a rehabilitation pro- gramme for chronic pain/fatigue." (p.100) | Qualitative - semi structured interviews | Various chronic pain conditions |
| Bastemeijer et al.(55) | 2020 | Patient values in physiotherapy practice, a qualitative study | Netherlands | "[…] to describe the aspects of physiotherapy practice that people with musculoskeletal pain value in high‐quality care." (p.2) | Qualitative - semi structured interviews | Low back / neck / shoulder pain |
| Bath et al.(56) | 2011 | Patient and referring health care provider satisfaction with a physiotherapy spinal triage assessment service | Canada | "[…] to evaluate patient and referring care provider satisfaction associated with a spinal triage service delivered by PTs in collaboration with orthopedic surgeons." (p.2) | Mixed methods - surveys | Low back pain |
| Battista et al.(57) | 2022 | Giving an account of patients' experience: A qualitative study on the care process of hip and knee osteoarthritis | Italy | "[…] explored the experience of people with OA about the care process they received in Italy." (p.1141) | Qualitative - semi structured interviews | Hip and knee osteoarthritis |
| Battista et al.(126) | 2021 | Experience of the COVID-19 pandemic as lived by patients with hip and knee osteoarthritis: an Italian qualitative study | Italy | "To examine the experience of the COVID-19 pandemic as lived by people with hip and knee osteoarthritis, in Italy." (p.1) | Qualitative - semi structured interviews | Hip and knee osteoarthritis |
| Bee et al.(127) | 2016 | Managing chronic widespread pain in primary care: a qualitative study of patient perspectives and implications for treatment delivery | England and Scotland | "[…] to explore participants’ illness and treatment experiences, with a view to understanding their potential influences on intervention acceptability." (p.2) | Qualitative - semi structured interviews | Chronic widespread pain |
| Benjaminsson et al.(111) | 2007 | Recurrent low back pain: relapse from a patient perspective | Sweden | "[…] to explore and describe how patients with recurrent low back pain perceive and respond to the recurrence of pain." (p.641) | Qualitative - semi structured interviews | Low back pain |
| Bibby(58) | 2006 | The management of chronic neck pain – A retrospective survey of the patient journey using in-depth semi-structured interviews | England | "[…] explores the experiences of patients from a specified locality who suffer with chronic neck pain." (p.25) | Qualitative - semi structured interviews | Chronic neck pain |
| Bin Sheeha et al.(128) | 2020 | Patients' experiences and satisfaction at one year following primary total knee arthroplasty: A focus-group discussion | England | "[…] to understand patient experiences, out- comes and satisfaction post-TKA" (p.2) | Qualitative - focus group | Knee osteoarthritis - post total knee arthroplasty |
| Bravo et al.(94) | 2018 | Experiences from group basic body awareness therapy by patients suffering from fibromyalgia: A qualitative study | Spain | "[…]to obtain a deeper understanding of how patients with fibromyalgia experienced and described movement awareness through participating in a Basic Body Awareness Group Therapy." (p.3) | Qualitative - interviews and focus group | Fibromyalgia |
| Brown et al.(142) | 2022 | In a pandemic that limits contact, can videoconferencing enable interdisciplinary persistent pain services and what are the patient’s perspectives? | Australia | "To explore patients' thoughts and satisfaction with using videoconferencing during the COVID-19 pandemic." (p.418) | Mixed methods - surveys | Not specified |
| Bunzli et al.(59) | 2016 | Patient perspectives on participation in cognitive functional therapy for chronic low back pain | Ireland and Australia | "[…] to investigate participants’ experience of cognitive functional therapy (CFT) by comparing participants who reported differing levels of improvement after participation in CFT, potentially yielding insight into the implementation of this approach." (p.1397) | Qualitative - semi structured interviews | Low back pain |
| Calner et al.(82) | 2021 | Physiotherapy treatment experiences of persons with persistent musculoskeletal pain: A qualitative study | Sweden | "[…] to explore and describe the physiotherapy treatment experiences of persons with persistent musculoskeletal pain." (p.29) | Qualitative - semi structured interviews | Various musculoskeletal conditions |
| Calner et al.(85) | 2017 | “I know what I want but I’m not sure how to get it”—Expectations of physiotherapy treatment of persons with persistent pain | Sweden | "[…] to explore and describe the expectations people with persistent back, neck, or shoulder pain have prior to physiotherapy treatment." (p.199) | Qualitative - semi structured interviews | Low back / neck / shoulder pain |
| Campbell et al.(116) | 2001 | Why don’t patients do their exercises? Understanding non-compliance with physiotherapy in patients with osteoarthritis of the knee | England | "To understand reasons for compliance and non-compliance with a home-based exercise regimen by patients with osteoarthritis of the knee." (p.132) | Qualitative - semi structured interviews | Knee osteoarthritis |
| Carmona-Terés et al.(60) | 2017 | Understanding knee osteoarthritis from the patients’ perspective: a qualitative study | Spain | "To identify current practice and advice of primary care professionals from the patients’ perspective in order to achieve a participative design of a complex intervention based on coaching psychology [...] To further understand the experiences, perceptions, cognitive evaluation, values, emotions, beliefs and coping strategies of patients with knee OA and the influence of all these factors in the Self-management of this condition." (p.2) | Qualitative - semi structured interviews | Knee osteoarthritis |
| Cederbom et al.(61) | 2020 | The perceptions of older adults living with chronic musculoskeletal pain about participating in an intervention based on a behavioral medicine approach to physical therapy. | Norway | "[…] to describe the perceptions of older people of participating in a 12-week home-based behavioral medicine approach to physical therapy […]" (p.1119) | Qualitative - semi structured interviews | Musculoskeletal pain |
| Cederbom et al.(145) | 2014 | The importance of a daily rhythm in a supportive environment – promoting ability in activities in everyday life among older women living alone with chronic pain | Sweden | "[…] to explore how older women living alone with chronic musculoskeletal pain, describe their ability in performing activities in everyday life and what could promote their ability in activities in everyday life as well as their perceived meaning of a changed ability to perform activities in everyday life." (p.2051) | Qualitative - semi structured interviews | Musculoskeletal pain |
| Che Hasan et al.(98) | 2021 | Perspectives of ESCAPE-Pain programme for older people with knee osteoarthritis in the community setting. | Malaysia | "[…] to understand how OA is currently treated in primary care setting and to explore the perspectives of healthcare professionals and patients on the acceptability and feasibility of a structured self–management programme that involves community approach." (p.2) | Qualitative - semi structured interviews | Knee osteoarthritis |
| Churchill et al.(120) | 2020 | A qualitative dominant mixed methods exploration of novel educational material for patients considering total knee arthroplasty | Canada | "[…] to garner end users’ (patients) experiences and perspectives regarding the content and clarity of videos and to better understand their potential impact on patient’s health behaviour." (p.2) | Mixed methods - semi structured interviews and survey | Knee osteoarthritis |
| Cook et al.(62) | 2000 | Active rehabilitation for chronic low back pain: The patients' perspective | England | "[…] to gain an in-depth understanding of individual patients’ experiences of chronic low back pain (CLBP), and active rehabilitation." (p.61) | Qualitative - semi structured interviews | Low back pain |
| Cooper et al.(122) | 2017 | Exploring peer-mentoring for community dwelling older adults with chronic low back pain: a qualitative study. | Scotland | "[…] to explore the perceptions of community dwelling older adults with CLBP, physiotherapists, and potential peer volunteers in relation to peer-mentoring for CLBP self-management following discharge from physiotherapy." (p.139) | Qualitative - semi structured interviews | Low back pain |
| Cooper et al.(50) | 2009 | Patients’ perceptions of self-management of chronic low back pain - evidence for enhancing patient education and support | Scotland | "To explore the extent to which physiotherapy facilitated chronic low back pain (CLBP) patients to self-manage following discharge, and to explore patients’ perceptions of their need for self-management interventions or support and their preferences in terms of delivery." (p.43) | Qualitative - semi structured interviews | Low back pain |
|  |  |  |  |  |  |  |
| Cooper et al.(63) | 2008 | Patient-centredness in physiotherapy from the perspective of the chronic low back pain patient | England and Scotland | "[…] to define patient-centredness, in the context of physiotherapy for CLBP, from the patient’s perspective." (p.245) | Qualitative - semi structured interviews | Low back pain |
| Corey et al.(64) | 2022 | Physiotherapists' role in physical activity promotion: Qualitative reflections of patients and providers. | United States | "[…] to examine strategies to promote physical activity in physiotherapy, and to capture patients’ reflections on the integration of community-based programs as a resource within these strategies." (p.3) | Qualitative - semi structured interviews | Various musculoskeletal and neurologic conditions |
| Cronström et al.(100) | 2019 | ‘I would never have done it if it hadn’t been digital’: a qualitative study on patients’ experiences of a digital management programme for hip and knee osteoarthritis in Sweden | Switzerland | "[…] to investigate the patients’ experiences of using a digital management programme for hip and knee OA." (p.2) | Qualitative - semi structured interviews | Knee and hip osteoarthritis |
| Daulat(133) | 2016 | A pragmatic randomized controlled trial to compare a novel group physiotherapy programme with a standard group exercise programme for managing chronic low back pain in primary care | England | "To compare a novel group physiotherapy exercise programme with a standard programme for managing chronic low back pain (CLBP) in primary care." (p.97) | Mixed methods - randomized controlled study and focus group | Low back pain |
| Davis et al.(86) | 2002 | Barriers to managing chronic pain of older adults with arthritis. | United States | "To explore barriers to pain management experienced by older adults with arthritis, identify themes, and develop a theoretical model of relationships among the themes." (p.121) | Qualitative - focus group | Various arthritis diagnoses |
| Escolar-Reina et al.(123) | 2010 | How do care-provider and home exercise program characteristics affect patient adherence in chronic neck and back pain: a qualitative study | Spain | "[…] to explore how the intrinsic characteristics of home-based exercise programme or care provider' style in clinical settings affects chronic neck or low back pain patients' adherence to prescribed exercise." (p.2) | Qualitative - focus group | Neck and low back pain |
| Ezzat et al.(99) | 2022 | “Much better than I thought it was going to be”: Telehealth delivered group-based education and exercise was perceived as acceptable among people with knee osteoarthritis | Australia | "[…] to understand patient perceived acceptability of participating in a telehealth delivered group-based education and exercise-therapy program for knee osteoarthritis." (p.2) | Qualitative - semi structured interviews | Knee osteoarthritis |
| Feldman et al.(65) | 2021 | Patients' prior perceptions and expectations of the Enhanced Transtheoretical Model Intervention for chronic low back pain - A qualitative study | Israel | "[…] to explore CLBP patients' perceptions and expectations of the ETMI method prior to their first consultation with physiotherapist." (p.372) | Qualitative - semi structured interviews | Low back pain |
| Fritz et al.(144) | 2021 | Perceptions of telehealth physical therapy among patients with chronic low back pain | United States | "[…] to describe the perceptions of telehealth delivery of physical therapy among patients with chronic LBP." (p.259) | Quantitative - survey | Low back pain |
| Furness et al.(66) | 2020 | A qualitative exploration of experiences of physiotherapy among people with fibromyalgia in the United Kingdom | England | "[…] to investigate the experiences of physiotherapy from the perspective of people with fibromyalgia in the United Kingdom, using a qualitative survey design." (p.38) | Mixed methods - surveys | Fibromyalgia |
| Geuens et al.(129) | 2019 | Mobile health features supporting self-management behavior in patients with chronic arthritis: mixed-methods approach on patient preferences | Belgium | "[…] to determine the preference of features for an mHealth app to support self-management behavior in patients with CA. In addition, we aimed to explore the motives behind these ratings." (p.1) | Mixed methods - semi structured interviews and survey | Chronic arthritis |
| Gillis et al.(135) | 2014 | Physiotherapy extended-role practitioner for individuals with hip and knee arthritis: patient perspectives of a rural/urban partnership. | Canada | "To explore the perspectives of people with hip and knee arthritis regarding a physiotherapy extended-role practitioner (ERP) model of care in a rural setting." (p.25) | Qualitative - semi structured interviews | Knee and hip osteoarthritis |
| Goldsmith et al.(67) | 2017 | The importance of informational, clinical and personal support in patient experience with total knee replacement: a qualitative investigation | Canada | "[…] to improve our understanding of patient experience and patient satisfaction following TKA surgery." (p.2) | Qualitative - semi structured interviews | Knee osteoarthritis - post total knee arthroplasty |
| Hilberdink et al.(124) | 2019 | Supervised group exercise in axial spondyloarthritis: patients’ satisfaction and perspective on evidence-based enhancements | Netherlands | "[…]to describe axial SpA patients’ satisfaction with current SGE and perspective on potential evidence-based SGE enhancements." (p.829) | Quantitative - survey | Axial spondyloarthritis |
| Hills et al.(68) | 2005 | Satisfaction with outpatient physiotherapy: Focus groups to explore the views of patients with acute and chronic musculoskeletal conditions | England | "We explored the factors that affect patients’ satisfaction with musculoskeletal outpatient physiotherapy." (p.1) | Qualitative - focus group | Various musculoskeletal conditions |
| Hinman et al.(91) | 2017 | “Sounds a bit crazy, but it was almost more personal:” a qualitative study of patient and clinician experiences of physical therapist– prescribed exercise for knee osteoarthritis via skype | Australia | "To explore the experience of patients and physical therapists with Skype for exercise management of knee osteoarthritis (OA)."(p.1834) | Qualitative - semi structured interviews | Knee osteoarthritis |
| Hinman et al.(69) | 2016 | Physical therapists, telephone coaches, and patients with knee osteoarthritis: qualitative study about working together to promote exercise adherence | Australia | "[…] to explore how stakeholders (physical therapists, telephone coaches, and patients) experienced, and made sense of, being involved in an integrated program of physical therapist–supervised exercise and telephone coaching for people with knee OA." (p.479) | Qualitative - semi structured interviews | Knee osteoarthritis |
| Holopainen et al.(70) | 2020 | Patients’ conceptions of undergoing physiotherapy for persistent low back pain delivered in Finnish primary healthcare by physiotherapists who had participated in brief training in cognitive functional therapy | Finland | "To explore the conceptions of patients with persistent low back pain (LBP) of undergoing physiotherapy delivered in Finnish primary healthcare by physiotherapists who had participated in brief training in Cognitive Functional Therapy (CFT)." (p.3388) | Qualitative - semi structured interviews | Low back pain |
| Jensen et al.(71) | 2021 | Patients’ expectations of physiotherapeutic treatment for long-term side effects after cancer: a qualitative study | Danemark | "[…] to explore the expectations of physiotherapeutic treatment of long-term side effects (LTSEs) after cancer among patients treated in physiotherapy clinics." (p.1) | Qualitative - semi structured interviews | Cancer related pain |
| Joelsson et al.(101) | 2017 | Patients with chronic pain may need extra support when prescribed physical activity in primary care: a qualitative study. | Sweden | "[…] to describe the experiences of and thoughts about receiving a prescription for physical activity of people with chronic musculoskeletal pain." (p.64) | Qualitative - semi structured interviews | Various musculoskeletal conditions |
| Josefsson et al.(51) | 2012 | Sexual health in patients with rheumatoid arthritis - experiences, needs and communication with health care professionals | Sweden | "[…] to explore the experiences and views of patients concerning the impact of RA on their sexual health, the possible impact of physiotherapy interventions, and communication in clinical situations." (p.1) | quantitative - survey | Rheumatoid arthritis |
| Joyce et al.(87) | 2022 | Beyond the pain: A qualitative study exploring the physical therapy experience in patients with chronic low back pain. | United States | "To describe the experience of physical therapy in a predominantly low-income and minority population with cLBP." (p.1) | Qualitative - semi structured interviews | Low back pain |
| Kamper et al.(112) | 2018 | What do patients with chronic spinal pain expect from their physiotherapist? | Canada | "[…] to describe LBP patients’ expectations of physiotherapy." (p.36) | Quantitative - survey | Low back pain |
| Kemp(52) | 1999 | Personal perceptions of the community service needs of persons with spinal injuries (stage four of the thesis: charting a parallel course - meeting the community service needs of persons with spinal injuries) | Australia | "[…] to determine service priorities based upon persons with spinal injuries' personal perceptions of need." (p.25) | Mixed methods - semi structured interviews and survey | Spinal cord injury |
| Larmer et al.(102) | 2014 | Patient reported benefits of hydrotherapy for arthritis | New-Zealand | "[…] to explore the perceived benefits of hydrotherapy from a patient’s perspective." (p.89) | Qualitative - semi structured interviews and focus group | Knee and hip osteoarthritis |
| Larsson et al.(130) | 2019 | The feasibility of gym-based exercise therapy for patients with persistent neck pain | Sweden | "[…] to evaluate whether supervised exercises addressing the most often impaired functions in these patients might be a feasible and helpful intervention for patients with persistent neck pain. A secondary aim was to investigate the patients’ and physical therapists’ experiences and perspectives of this exercise model." (p.2) | Mixed methods - surveys | Neck pain |
| Lawford et al.(72) | 2018 | “I was really sceptical...But it worked really well”: a qualitative study of patient perceptions of telephone-delivered exercise therapy by physiotherapists for people with knee osteoarthritis | Australia | "[…] to explore peoples' perceptions of exercise therapy delivered by physiotherapists via telephone for their knee OA." (p.741) | Qualitative - semi structured interviews | Knee osteoarthritis |
| Lawford et al.(143) | 2017 | Consumer perceptions of and willingness to use remotely delivered service models for exercise management of knee and hip osteoarthritis: a cross-sectional survey. | Australia | "To investigate the perceptions of people with hip and/or knee osteoarthritis (OA) about the remote delivery of exercise therapy by a physical therapist." (p.667) | Quantitative - survey | Knee and hip osteoarthritis |
| Ledingham et al.(103) | 2019 | Exercise adherence: beliefs of adults with knee osteoarthritis over 2 years | United States | "[…] to explore experiences, feelings, and perspectives related to long-term adherence to exercise among adults with painful KOA participating in a 2-year RCT, and identify factors that influenced long-term adherence to exercise." (p.1373) | Qualitative - semi structured interviews | Knee osteoarthritis |
| LePage et al.(53) | 2016 | What do patients want? A needs assessment of vulvodynia patients attending a vulvar diseases clinic | Canada | "[…] to identify unmet needs among localized provoked vulvodynia patients." (p.242) | Qualitative - semi structured interviews | Vulvodynia |
| Liddle et al.(83) | 2007 | Chronic low back pain: Patients' experiences, opinions and expectations for clinical management | Ireland | "To explore the experiences, opinions and treatment expectations of chronic low back pain (LBP) patients in order to identify what components of treatment they consider as being of most value." (p.1899) | Qualitative - focus group | Low back pain |
| Louw et al.(104) | 2018 | The experiences and beliefs of patients with complex regional pain syndrome: An exploratory survey study | United States and Canada | "To determine the beliefs and describe the health care experiences of patients with complex regional pain syndrome." (p.104) | Quantitative - survey | Complex regional pain syndrome |
| Lovo et al.(137) | 2019 | Experience of patients and practitioners with a team and technology approach to chronic back disorder (CBD) management | Canada | "[…] to describe the experience of health care providers and patients who participated in a team and technology model of care for management of CBD." (p.857) | Mixed methods - semi structured interviews and survey | Low back pain |
| Maki et al.(119) | 2021 | An exploration of experiences and beliefs about low back pain with Arab Muslim patients | Bahrain | "[…] to explore the experiences and beliefs of Arab Muslim patients with low back pain (LBP) in Bahrain." (p.1) | Qualitative - focus group | Low back pain |
| Mannerkorpi et al.(105) | 2003 | Physiotherapy group treatment for patients with fibromyalgia—an embodied learning process | Sweden | "To study how patients with fibromyalgia (FM) experienced physiotherapy group treatment comprising pool exercise and education." (p.1372) | Qualitative - semi structured interviews | Fibromyalgia |
| Martensson et al.(92) | 2005 | Experiences of a primary health care rehabilitation programme. A focus group study of persons with chronic pain | Sweden | "[…] to describe participants’ experiences of a rehabilitation programme for persons with chronic pain." (p.985) | Qualitative - focus group | Various chronic pain conditions |
| May(73) | 2007 | Patients’ attitudes and beliefs about back pain and its management after physiotherapy for low back pain | United Kingdom | "[…] explored patients’ perspective and attitudes about back pain and its management using an explorative qualitative approach." (p.127) | Qualitative - semi structured interviews | Low back pain |
| May(74) | 2001 | Part 2: An explorative, qualitative study into patients’ satisfaction with physiotherapy | United Kingdom | "To describe the aspects of physiotherapy care which back pain patients consider important." (p.10) | Qualitative - semi structured interviews | Low back pain |
| Medina-Mirapeix et al.(106) | 2009 | Personal characteristics influencing patients’ adherence to home exercise during chronic pain: a qualitative study | Spain | "To identify the beliefs and perceptions of patients with chronic neck and low back pain that influence adherence to home exercise during exacerbation and/or remission of pain." (p.347) | Qualitative - focus group | Neck and low back pain |
| Mengshoel et al.(107) | 2021 | ‘It takes time, but recovering makes it worthwhile’- A qualitative study of longterm users’ experiences of physiotherapy in primary health care | Norway | "[…] to explore how patients in a Norwegian context make sense of their long-term use of physiotherapy." (p.6) | Qualitative - semi structured interviews | Various musculoskeletal conditions |
| Mengshoel et al.(139) | 2008 | Clinical significance of specific spinal mobilization for patients with ankylosing spondylitis evaluated by quantitative assessments and patient interviews | Norway | "To assess individual responses to specific spinal mobilization (SSM) in terms of spinal mobility, perceived stiffness, pain and activities of daily living (ADL) in patients with Ankylosis Spondylitis (AS), and whether and how interviews supported or supplemented the quantitative results." (p.355) | Mixed methods - clinical outcome measures, surveys and semi-structured interviews | Ankylosing spondylitis |
| Moffat et al.(131) | 2019 | Barriers and solutions to participation in exercise for moderately disabled people with multiple sclerosis not currently exercising: a consensus development study using nominal group technique | Scotland | "[…] to explore consensus on the barriers and solutions to exercise for people with MS living in Scotland." (p.2775) | Qualitative - focus group | Multiple sclerosis |
| Moore et al.(75) | 2020 | Therapeutic alliance facilitates adherence to physiotherapy-led exercise and physical activity for older adults with knee pain: a longitudinal qualitative study | United Kingdom | "[…] to investigate participants’ experiences of treatment, and barriers and facilitators to exercise and general physical activity behaviour in the longer term." (p.46) | Qualitative - semi structured interviews | Knee osteoarthritis |
| Nair et al.(138) | 2016 | Self-reported barriers to healthcare access for rheumatoid arthritis patients in rural and northern Saskatchewan: a mixed methods study | Canada | "[…] to identify potential barriers for access to medical and allied health services from the perspective of rural and Northern Saskatchewan rheumatoid arthritis (RA) patients." (p.243) | Qualitative - structured interviews | Rheumatoid arthritis |
| Nees et al.(134) | 2020 | Multidisciplinary Pain Management of Chronic Back Pain: Helpful Treatments from the Patients' Perspective. | Germany | "[…] to assess the patients’ perceived helpfulness of different treatment modalities, the influence of sociodemographic characteristics on the patient’s perspective and whether treatment outcomes are affected by helpfulness ratings." (P.1) | Quantitative - survey | Low back pain |
| Nøst et al.(115) | 2019 | A lifebuoy’ and ‘a waste of time’: patients’ varying experiences of multidisciplinary pain center treatment- a qualitative study | Norway | "[…] to investigate variation in patients’ experiences of attending individual outpatient multidisciplinary treatment at pain centers in Norway." (p.1) | Qualitative - semi structured interviews | Various musculoskeletal and neurologic conditions |
| Odole et al.(88) | 2022 | Patients’ satisfaction with physiotherapy management of chronic mechanical neck pain in physiotherapy departments of public hospitals in Ibadan, Nigeria: A mixed-method study | Nigeria | "[…] determining patients’ satisfaction level with physiotherapy in the management of chronic mechanical neck pain (CMNP) in physiotherapy departments of the 3 public hospitals in Ibadan, Nigeria." (p.1) | Mixed methods - survey and focus group | Neck pain |
| Øien et al.(76) | 2011 | Communication as negotiation processes in long-term physiotherapy: a qualitative study | Norway | "[…] to describe communicative patterns about change in demanding physiotherapy treatment situations." (p.53) | Qualitative - semi structured interviews | Neck and low back pain |
| Øien et al.(140) | 2007 | Narratives of embodied experiences – Therapy processes in Norwegian psychomotor physiotherapy | Norway | "[…] to explore patients’ narratives of embodied experiences through treatment courses of Norwegian psychomotor physiotherapy (NPMP)." (p.31) | Qualitative - interviews, field observations and video-recording | Low back pain |
| Palazzo et al.(108) | 2016 | Barriers to home-based exercise program adherence with chronic low back pain: Patient expectations regarding new technologies | France | "To assess views of patients with chronic low back pain (cLBP) concerning barriers to homebased exercise program adherence and to record expectations regarding new technologies." (p.107) | Qualitative - semi structured interviews | Low back pain |
| Petursdottir et al.(77) | 2010 | Facilitators and barriers to exercising among people with osteoarthritis: a phenomenological study | Iceland | "[…] to increase knowledge and understanding of the experience of exercising among individuals with OA and to determine what they perceive as facilitators and barriers to exercising." (p.1014) | Qualitative - semi structured interviews | Various osteoarthritis conditions |
| Plank et al.(113) | 2021 | Exploring expectations and perceptions of different manual therapy techniques in chronic low back pain: a qualitative study | United Kingdom | "To explore expectations and perceptions of MT techniques in people with CLBP." (p.1) | Qualitative - semi structured interviews | Low back pain |
| Reid et al.(141) | 2014 | Physiotherapy management of knee and hip osteoarthritis: a survey of patient and medical practitioners’ expectations, experiences and perceptions of effectiveness of treatment | New-Zealand | "[…] to investigate self-reported behaviour, experiences, expectations and perceptions of individuals with OA of the hip and knee GPs, and orthopaedic surgeons with regards to physiotherapy referral and management." (p.118) | Quantitative - survey | Knee and hip osteoarthritis |
| Selten et al.(109) | 2016 | Reasons for treatment choices in knee and hip osteoarthritis: a qualitative study | Netherlands | "[…] to identify the reasons for treatment choices of patients with knee or hip OA in primary and secondary care." (p.1261) | Qualitative - semi structured interviews | Knee and hip osteoarthritis |
| Skolasky et al.(132) | 2022 | Identifying perceptions, experiences, and recommendations of telehealth physical therapy for patients with chronic low back pain: a mixed methods survey | United States | "To describe concerns, advantages, and disadvantages encountered in an evidence-based physical therapy (PT) program for persons with chronic low back pain (CLBP) delivered by telehealth." (p.1935) | Mixed methods - semi structured interviews and survey | Low back pain |
| Slade et al.(78) | 2009 | People with non-specific chronic low back pain who have participated in exercise programs have preferences about exercise: a qualitative study | Australia | "[…] to determine the experience of exercise programs by people with chronic low back pain." (p.115) | Qualitative - focus group | Low back pain |
| Slade et al.(79) | 2009 | Stigma experienced by people with nonspecific chronic low back pain: a qualitative study | Australia | "To determine participant experience of exercise programs for nonspecific chronic low back pain (NSCLBP)." (p.143) | Qualitative - focus group | Low back pain |
| Spitaels et al.(95) | 2016 | Barriers for guideline adherence in knee osteoarthritis care: A qualitative study from the patients’ perspective | Belgium | "[…] to investigate barriers and facilitators in current care of knee OA from the patients’ perspective." (p.166) | Qualitative - semi structured interviews | Knee osteoarthritis |
| Stenner et al.(54) | 2016 | Exercise prescription for non-specific chronic low back pain (NSCLBP): a qualitative study of patients' experiences of involvement in decision making. | United Kingdom | "To understand the treatment decision making experiences, information and decision support needs of patients with NSCLBP who have been offered exercise as part of their management plan." (p.339) | Qualitative - semi structured interviews | Low back pain |
| Stephens et al.(96) | 2020 | “It’s just like a needle going into my hip, basically all of the time”. The experiences and perceptions of patients with Greater Trochanteric Pain syndrome in the UK National Health Service | United Kingdom | "[…] to provide insight into the experiences and perceptions of patients suffering with GTPS." (p.1) | Qualitative - semi structured interviews | Great trochanteric pain syndrome |
| Swärdh et al.(90) | 2020 | “A necessary investment in future health”: perceptions of physical activity maintenance among people with rheumatoid arthritis | Sweden | "[…] to describe perceptions of PA maintenance during the second year of an outsourced 2-year support program among people with RA." (p.2144) | Qualitative - semi structured interviews | Rheumatoid arthritis |
| Swärdh et al.(89) | 2008 | Views on exercise maintenance: variations among patients with rheumatoid arthritis | Sweden | "[…] to explore and describe ways of understanding exercise maintenance among individuals with RA who had already started to exercise." (p.1049) | Qualitative - semi structured interviews | Rheumatoid arthritis |
| Teo et al.(80) | 2021 | Patient experiences with physiotherapy for knee osteoarthritis in Australia-a qualitative study. | Australia | "[…] to explore the experiences of people who had recently received physiotherapy care for their knee OA in Australia and how these experiences aligned with the national Clinical Care Standard for knee OA." (p.1) | Qualitative - semi structured interviews | Knee osteoarthritis |
| Thorne et al.(81) | 2004 | Health care communication issues in fibromyalgia: an interpretive description | Canada | "To portray how individuals with fibromyalgia (FM) describe interactions with health care providers and to highlight patterns of communication that were perceived as helpful or unhelpful during these encounters." (p.31) | Qualitative - semi structured interviews | Fibromyalgia |
| Toye et al.(117) | 2012 | ‘I can’t see any reason for stopping doing anything, but I might have to do it differently’ – restoring hope to patients with persistent non-specific low back pain – a qualitative study | United Kingdom | "To explore the differences in narrative between patients with persistent non-specific low back pain (PLBP) who benefited from a pain management programme, and those who did not benefit." (p.894) | Qualitative - semi structured interviews | Low back pain |
| Veenhof et al.(114) | 2006 | Active involvement and long-term goals influence longterm adherence to behavioural graded activity in patients with osteoarthritis: a qualitative study | Netherlands | "[…] to investigate which factors explain the difference, after a behavioural graded activity program, between patients who successfully integrate activities in their daily lives and those who do not succeed in integrating activities in their daily lives." (p.273) | Qualitative - semi structured interviews | Knee and hip osteoarthritis |
| Wallis et al.(118) | 2019 | Perceptions about participation in a 12-week walking program for people with severe knee osteoarthritis: a qualitative analysis | Australia | "To explore the perceptions of people with severe knee osteoarthritis and increased cardiovascular risk about participating in a walking program." (p.779) | Qualitative - semi structured interviews | Knee osteoarthritis |
| Wijma et al.(84) | 2018 | What is important in transdisciplinary pain neuroscience education? A qualitative study. | Netherlands | "[…] to explore the experiences in patients with non-specific chronic pain." (p.2181) | Qualitative - semi structured interviews and focus group | Non-specific chronic pain |
| Willett et al.(97) | 2021 | Utilising the perspectives of patients with lower-limb osteoarthritis on prescribed physical activity to develop a theoretically informed physiotherapy intervention | England | "[…] to use knowledge of identified barriers and facilitators to physiotherapy prescribed PA (during treatment and post- discharge) to develop a theoretically informed intervention to optimise adherence to PA for patients with lower- limb OA during treatment and post-discharge." (p.1) | Qualitative - semi structured interviews | Knee and hip osteoarthritis |
| Wilson et al.(93) | 2016 | Psychologically informed physiotherapy for chronic pain: patient experiences of treatment and therapeutic process | United Kingdom | "This study aimed to investigate patients’ beliefs about, and experiences of, this type of treatment, and helpful and unhelpful experiences." (p.98) | Qualitative - semi structured interviews | Non-specific chronic pain |
| Winter Di Cola et al.(121) | 2014 | Patients’ perceptions of navigating ‘‘the system’’ for arthritis management: are they able to follow our recommendations? | Canada | "To understand whether a visit to a Hip/Knee Arthritis Assessment Centre (AC), where non-surgical candidates with arthritis are directed toward community resources and provided with a conservative treatment ‘‘prescription,’’ contributes to patients’ self-management and ability to access community resources." (p.264) | Qualitative - semi structured interviews and focus group | Knee and hip osteoarthritis |
| Withall et al.(110) | 2016 | Physical activity engagement in early rheumatoid arthritis: a qualitative study to inform intervention development | United Kingdom | "To explore patient’s views on approaches to delivering PA programmes and inform a programme to maximise functional ability through long-term engagement with PA." (p.1) | Qualitative - focus group | Rheumatoid arthritis |
